# The Importance of Conserving Biodiversity Outside of Protected Areas in Mediterranean Ecosystems

**DOI:** 10.1371/journal.pone.0014508

**Published:** 2011-01-07

**Authors:** Robin L. Cox, Emma C. Underwood

**Affiliations:** 1 The Nature Conservancy, San Francisco, California, United States of America; 2 Department of Environmental Science and Policy, University of California Davis, Davis, California, United States of America; University of Western Ontario, Canada

## Abstract

Mediterranean-type ecosystems constitute one of the rarest terrestrial biomes and yet they are extraordinarily biodiverse. Home to over 250 million people, the five regions where these ecosystems are found have climate and coastal conditions that make them highly desirable human habitats. The current conservation landscape does not reflect the mediterranean biome's rarity and its importance for plant endemism. Habitat conversion will clearly outpace expansion of formal protected-area networks, and conservationists must augment this traditional strategy with new approaches to sustain the mediterranean biota. Using regional scale datasets, we determine the area of land in each of the five regions that is protected, converted (e.g., to urban or industrial), impacted (e.g., intensive, cultivated agriculture), or lands that we consider to have conservation potential. The latter are natural and semi-natural lands that are unprotected (e.g., private range lands) but sustain numerous native species and associated habitats. Chile has the greatest proportion of its land (75%) in this category and California-Mexico the least (48%). To illustrate the potential for achieving mediterranean biodiversity conservation on these lands, we use species-area curves generated from ecoregion scale data on native plant species richness and vertebrate species richness. For example, if biodiversity could be sustained on even 25% of existing unprotected, natural and semi-natural lands, we estimate that the habitat of more than 6,000 species could be represented. This analysis suggests that if unprotected natural and semi-natural lands are managed in a manner that allows for persistence of native species, we can realize significant additional biodiversity gains. Lasting biodiversity protection at the scale needed requires unprecedented collaboration among stakeholders to promote conservation both inside and outside of traditional protected areas, including on lands where people live and work.

## Introduction

Geographic regions with a mediterranean climate form one of the rarest of the Earth's thirteen terrestrial biomes, covering a mere 2% of the Earth's land surface [1], [2]. What the mediterranean biome lacks in size is compensated by in biodiversity. Over 20% of Earth's known vascular plant taxa are found in this biome [2], [3], many of which are exceedingly rare and localized endemics [3]. Mediterranean regions, with their mild climate of cool wet winters and warm dry summers, are also home to millions of people and many of the world's major metropolitan areas, e.g., Rome (Italy), Santiago (Chile), Cape Town (South Africa), Los Angeles (USA), and Perth (Australia). As the world population continues to grow [4], the natural area of this diminutive biome will likely continue to shrink.

Although the mediterranean biome is widely recognized as a global conservation priority [5], [6], [7], only 4.3% of the biome is within formally protected reserves specifically designated for biodiversity protection (IUCN classes I-IV) [8], which is less than half of the accepted global protection goal for ecological systems [9]. Furthermore, protected areas that do exist are disproportionally concentrated in ‘left-over land’, ill-suited to economic uses, such as areas of high elevation and steep slopes in the Cape Region of South Africa [10].

Conserving native biota using formal protected areas alone is a strategy unlikely to succeed in mediterranean regions. These protected areas not only cover a very small proportion of the biome, they do not adequately represent its endemic biota [10], [11], particularly where there is high species turnover across the landscape. For example, the local diversity of burned, nutrient-poor heathlands of Australia's kwongan and South Africa's fynbos systems occurs at spatial scales in the order of 0.1 ha [1], [12]. To fully represent these highly localized species distribution patterns generally requires numerous reserves [13], an unlikely solution given constraints on conservation spending. In many mediterranean regions, endemic species persist only on small remnants of natural habitat separated by intensive agricultural (lands dedicated to cultivation) and urban areas, a distribution poorly suited for traditional protected areas due to high land values, complex ownership patterns, difficulties of managing ecological processes in close proximity to people, and other factors [14], [15]. The capacity of protected areas for conserving this unique biome is further complicated by the dependency of many mediterranean species on silvopastoral landscapes maintained by human interventions, for example, open-pasture endemics in the Mediterranean Basin [16], [17].

The future is likely to bring new challenges. Mediterranean regions are expected to be heavily impacted by climate change [18], compounding the already severe threats to native species. For example, over two-thirds of the endemic plant taxa of California may experience >80% range reductions within a century [19]. Only about half of the areas currently protected will still experience a mediterranean climate at the end of the century; moreover, nearly one third of the land projected to maintain a mediterranean climate has already been converted to human use (such as intensive agriculture), thus eliminating or limiting its potential to serve as climatic refugia for native species [18]. While there is debate on how best to adapt conservation strategies to climate change, there is wide agreement that more land must be protected rapidly, and that protection should be expanded outside of reserves [20], [21]. We use species-area relationships to estimate potential biodiversity gains that could be achieved by safeguarding native biodiversity within the regions' remaining natural and semi-natural, yet unprotected, lands.

## Methods

We defined the mediterranean biome using the 39 terrestrial ecoregions identified by the World Wildlife Fund [22] and used regional scale data on potential natural vegetation, current land use, and protected areas, with the exception of the Mediterranean Basin where we relied on global scale protected area data (see Underwood et al. 2009 for a full description). There have been previous regional, country, and within-country scale conservation planning studies assessing protected areas, land cover changes, and biodiversity across the mediterranean biome, e.g., in the Mediterranean Basin [17], [23], Chile [11] and South Africa [24], which have utilized regional scale taxonomic data, e.g., reptiles and amphibians of the Mediterranean Basin [25]. However, our objective was to utilize existing global taxonomic datasets to permit comparisons to be made across the five mediterranean regions.

To determine the amount of land that could potentially deliver biodiversity benefits across the mediterranean biome we calculated for each of the five regions the areal percentage of land in four land cover categories: ‘protected’ (IUCN categories I-VI which includes those protected areas managed mainly for recreation and sustainable use), ‘converted’, ‘impacted’, and lands with ‘conservation potential’. We define ‘converted’ lands as those that lack natural cover and are classified on land cover maps as urban, industrial, mines, or equivalent categories. We define ‘impacted’ lands as those classified as intensive agriculture including forest plantations, rotation crops, vineyards, orchards, and all other types of cultivation. Finally, lands with ‘conservation potential’ we define as those classified as a natural vegetation type (i.e., grasslands, woodlands, forests) located outside protected areas. We also refer to these as natural or semi-natural lands, recognizing that they may have invasive species, disrupted ecological processes, and human uses. Examples include private range lands, isolated native vegetation remnants within urban areas or cultivated zones, and other ‘working landscapes’ (i.e., areas of natural vegetation utilized for commodity production such as grazing, timber harvest, hunting and other economic activities that do not require large-scale and permanent removal of natural vegetation cover).

To estimate the number of species conserved under existing land cover status, we used the species-area relationship. In an effort to tailor the ‘z’ exponent of the species-area equation to the different taxa of mediterranean ecoregions, we used information on ecoregion area and the number of species of native plants [1], [26] and vertebrates (mammals, birds, reptiles, amphibians from the Wildfinder database) [27]. Within each region, we then estimate the number of species currently on protected lands (all IUCN categories I-VI) using the ‘z’ exponent specific to each taxa. The relationship between the number of species and area is one of the most widely recognized ecological patterns and it has been utilized in numerous other regional and global analyses to provide simple estimates of the gain in species with protection efforts [28], [29], [30]. It is, however, limited in that it assumes the random distribution of species. Existing protected areas in the mediterranean biome inadequately represent the diversity of habitat types across the biome [8], thus the estimates of species currently ‘protected’ are likely to be high.

We then use the species-area relationship in this study as a tool to illustrate the potential conservation returns that can be secured in natural and semi-natural lands that remain outside of protected areas. We explore a conservation scenario in which 25% of natural and semi- natural lands in each region are retained and managed in ways that allow native biodiversity to persist (e.g., appropriately-managed native forests and natural grasslands). Again, we use the species-area relationship to estimate the number of species by taxa, whose habitat could be conserved outside of formal protected areas.

## Results

**Figure 1:**
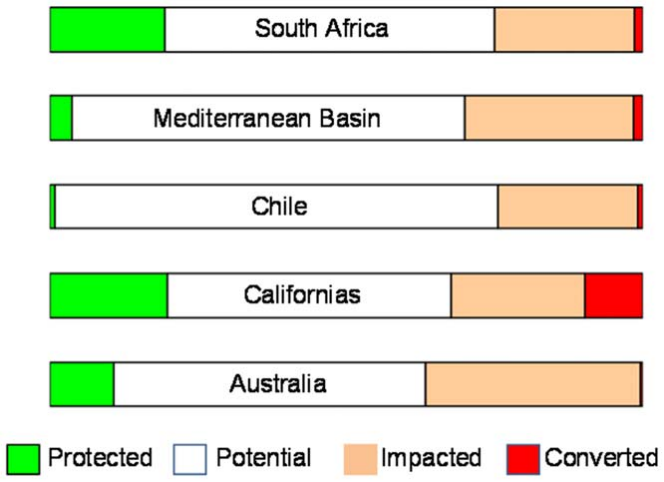
Proportional extent of land cover categories in each of the world's five mediterranean regions: protected (IUCN categories I-VI), converted (to urban), impacted (by intensive agriculture), and lands with conservation potential.

Using the four land cover categories across the whole biome, we found that 62% is within our potential conservation category, 7% is protected (IUCN categories I-VI, although only 4.3% specifically for biodiversity), 30% impacted, and 2% converted (Table 1). The mediterranean region of Chile has the greatest amount of potential conservation land proportional to area (75%), followed by the Mediterranean Basin (66%), South Africa (56%), Australia (53%), and the Californias (48%) (Fig. 1, Table 1, and see http://www.mediterraneanaction.net/ma_v2/about_biome/vegetation.jsp for maps).

**Table 1:** The area (km^2^) and proportion of land which is protected (IUCN categories I-VI), converted, impacted, or with conservation potential (see text for description of each class).

Region	Area (km^2^)	Protected area (IUCN I-VI) (km^2^ & proportion)	Area with conservation potential (km^2^ & proportion)	Impacted area (km^2^ & proportion)	Converted area (km^2^ & proportion)	No. native plants	No. of verts	Total no. of species
Australia	802,523	86,962 (11%)	422,010 (53%)	290,577 (36%)	2,974 (<1%)	8,000	754	8,754
Californias	176,425	34,955 (20%)	84,444 (48%)	39,934 (23%)	17,092 (10%)	4,300	487	4,787
Chile	148,408	1,370 (1%)	110,861 (75%)	35,076 (24%)	1,100 (1%)	2,400	198	2,598
Med Basin	2,077,131	77,411 (4%)	1,376,444 (66%)	592,299 (29%)	30,977 (1%)	25,000	920	25,920
South Africa	96,953	18,823 (19%)	54,015 (56%)	22,866 (24%)	1,249 (1%)	8,550	563	9,113
Total	3,301,440	219,521 (7%)	2,047,775 (62%)	980,752 (30%)	53,392 (2%)	48,250	2,922	51,172

Estimates of the number of native plants are from Cowling et al. (1996) and terrestrial vertebrates from World Wildlife Fund (2006).

**Figure 2:**
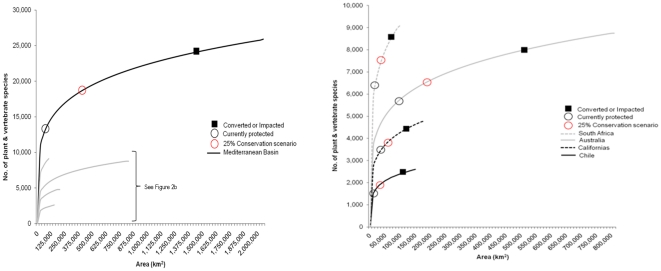
Comparison of species area curves for (a) the Mediterranean Basin; (b) South Africa, Australia, the Californias and Chile, based on the number of total plant and vertebrate species. The species currently protected (IUCN categories I-VI) are indicated (approximately) by a circle and the area that is converted or impacted is represented by a square. The potential lands are represented by the space in between these two points. The increase in species protected under a conservation scenario whereby 25% of natural and semi-natural landscapes in each region are managed for biodiversity conservation is shown by a red circle.

Notable differences in the species-area relationship exist among the five regions. For example, the long species-area curve of the Mediterranean Basin with its vast spatial extent and high number of native plants and vertebrates (25,000 and 920 respectively, Fig. 2a and Table 1) contrasts with the steep curve of South Africa with its high number of species in a small area (8,550 plants and 563 vertebrates, Fig. 2b and Table 1). Using the ‘z’ exponent specific to each taxa, ranging from a low of z = 0.125 for birds to a high of z = 0.315 for mammals (see Table 2 for other taxa), our model estimates that more than 34,000 plants currently occur on protected lands, ranging from 16,355 in the Mediterranean Basin to 1,311 in Chile (Table 2). Over 1,100 birds occur on protected lands, again with the highest number in the Mediterranean Basin (314) with similar numbers in Australia and South Africa (280). We also estimate 253, 125, and 285 mammals, amphibians, and reptiles respectively occur on protected areas across the mediterranean biome (Table 2).

**Table 2:** The number of native plants and vertebrates currently protected in each mediterranean region and those gained under a scenario whereby 25% of potential lands advance biodiversity conservation.

	No. of species currently protected						No. of species gained in 25% conservation scenario					
Region	Plants	Birds	Mamm.	Amph.	Rep.	Total	Plants	Birds	Mamm.	Amph.	Rep.	Total
Australia	6,006	280	44	41	125	6,496	648	29	12	5	33	727
Californias	3,490	243	65	17	37	3,852	219	15	10	1	5	250
Chile	1,311	73	9	3	5	1,402	634	34	15	2	7	692
Med Basin	16,355	314	79	35	64	16,846	3,996	74	56	10	41	4,177
South Africa	6,920	281	55	28	55	7,340	500	20	10	2	9	541
Total	34,082	1,191	253	125	285	35,936	5,997	172	103	20	95	6,387

We used species-area curves developed for each taxa; ‘z’ exponents were as follows: birds 0.125; mammals 0.315, amphibians 0.148, reptiles 0.293, and plants 0.129.

Assuming a future conservation scenario where 25% of current natural and semi-natural lands (i.e., those with conservation potential) in each region are managed in ways that allow native biodiversity to persist, we find an additional 5,997 plants and 390 vertebrates, ranging from 172 birds to 20 amphibians, could be represented on these additional lands (Fig. 2 and Table 2). Clearly, making currently unprotected natural and semi-natural lands work simultaneously for conservation and for people could deliver significant biodiversity conservation returns in the mediterranean biome, even under modest scenarios.

## Discussion

Although much of the biodiversity of the mediterranean biome is threatened, the amount of potential conservation land that remains is cause for optimism, presenting opportunities for less formal, yet enduring conservation approaches. Given the contrasting land use patterns within each of the biome's five regions, conservation strategies must be applied across a range of natural and semi-natural landscape configurations. For example, all regions have areas of concentrated fragmentation, especially in places where conservation competes poorly with privately held agriculture and urban areas, such Spain's Valencia region [31], South Africa's renosterveldt [32], [33], California's coastal scrub [34], and Australia's wheat belt [35]. In areas such as these, even small isolated patches are important refugia for endemic species and should be safeguarded, despite their vulnerability to exotic invasions, altered ecosystem function, and species loss. Fortunately, all five regions also support large blocks of unfragmented habitat, such as Australia's Great Western Woodlands [36] and California's Central Coast Ranges [37], that provide opportunities for conservation within the context of large, interconnected areas more resilient to disturbance.

There are conservation projects in all reaches of the biome that hold promise for safeguarding mediterranean biodiversity outside of protected areas, across the spectrum of landscape configurations, and we highlight three examples that could be adapted and leveraged more broadly across the biome to increase biodiversity outcomes: voluntary programs targeting native species and habitats on private, working landscapes in Europe and California, an emerging private lands conservation framework in Chile's matorral, and partnerships between the agricultural industry and conservation in South Africa.

First, in the Mediterranean Basin numerous endemic taxa persist only in ancient agrosilvopastoral landscapes, such as the cork oak-dominated *montado* of Portugal and *dehesa* of Spain [38]. These centuries-old agro-environmental systems produce economically valuable amenities such as cork, tourism and employment, while supporting numerous native wildlife and plant species, many of them endemic. In recognition of their biodiversity and cultural assets, these and other agro-environmental systems are considered high conservation priorities in Europe, and throughout the European Union governments pay farmers to voluntarily adapt their operations to benefit biodiversity and the environment [39]. Similarly, in California (USA), there are over four million hectares of native oak woodland ecosystems, representing approximately half of historic extent [40]. Most remaining woodlands are privately-owned working landscapes managed primarily for cattle grazing [41]. Like those of the Old World, these North American oak-dominated rangelands support hundreds of native species and provide countless human benefits including open space, watershed protection, forage provision, locally produced food, and preservation of a livestock ranching tradition valued by society [40], [42]. Recognizing that these private lands sustain one of California's most wildlife-rich ecosystems, governments and conservation organizations are expanding incentive-based programs that pay landowners for safeguarding these working landscapes, including both permanent legal agreements, e.g., conservation easements [43], and time-bound voluntary contracts, such as those included in the 2008 Farm Bill. The latter generated almost $54 million for farmers and ranchers in California (USA), much of it going to areas within the mediterranean biome [44], [45].

Second, in Chile, formal protection covers only one percent of the mediterarrean region, with the majority of the rest privately-owned [46], [47]. Private land conservation efforts are beginning to succeed; in 2009 Chile's first land trust was created to safeguard sensitive mediterranean habitats on private lands. Moreover, nongovernmental organizations are working in the Altos de Cantillana region of Chile's mediterranean region to develop the country's first legal framework for creating conservation easements and public-private land management agreements (see http://www.nature.org/wherewework/southamerica/chile/for details).

Third, agriculture is a major economic driver in all mediterranean regions, including a booming wine industry that is cause for ecological concern as native habitats are converted – impacting the native biota, ecological processes, water quality and aquatic health [32], [48]. Fortunately, in key wine-producing regions, there are emerging initiatives to pair agricultural expansion and land management practices with conservation. South Africa's Biodiversity and Wine Initiative (BWI), a partnership between the wine industry of the botanically renowned Cape Region and the country's conservation sector, has protected through private set-asides over 112,000 hectares of fynbos habitat since 2006, an area larger than the total wine footprint in the Cape Region (see http://www.bwi.co.za/). Similarly, growth of vineyards in California's premier coastal wine-growing regions has led to public debate over removal of natural vegetation, overproduction of wine grapes, and disappearance of foothill grasslands, oak woodlands, and riparian corridors [48]. These concerns, coupled with market competition, are generating tangible conservation outcomes, including private set-asides of natural habitat within and surrounding vineyards, local policies that restrict grading on steep slopes, better control of sediment and erosion near streams, and restoring stream flows for native salmonids while providing growers with a stable water supply [48], [49].

Unless the conservation community accelerates the pace of biodiversity conservation, many of the world's rare and endemic mediterranean systems will continue along their present trajectory of habitat conversion and degradation. The decline of these systems has a disproportionate impact on global biodiversity given their extraordinary endemism [1]. Renowned natural landscapes such as Australia's *Banksia* woodlands, the renosterveldt of South Africa's Cape Floristic Region [50], Baja California's coastal succulent scrub [51], Chile's matorral, and the unique agro-environmental systems of the Mediterranean Basin all face formidable conservation hurdles, given the demand on these and other mediterranean lands to support and house an additional hundreds of millions of people.

Fortunately, the large proportion of currently unprotected, natural and semi-natural land distributed across the five regions offers conservation a way forward. This analysis should not be interpreted to suggest that traditional formal protected areas are not essential. Rather, this analysis shows that if the remaining natural lands where people live and work are managed in a manner that allows for persistence of native species, we can realize significant additional biodiversity gains. To achieve that outcome, unprecedented collaboration will be needed among stakeholders within each region to develop and apply a range of conservation strategies that would include incentive-based voluntary programs, regulatory structures, legal frameworks, land use policies, and stable sources of funding. All of this will be challenging, but it is a challenge we collectively must meet.
